# Intestinal Microbiota and Vaccinations: A Systematic Review of the Literature

**DOI:** 10.3390/vaccines13030306

**Published:** 2025-03-12

**Authors:** Francesco Loddo, Pasqualina Laganà, Caterina Elisabetta Rizzo, Serena Maria Calderone, Bruno Romeo, Roberto Venuto, Daniele Maisano, Francesco Fedele, Raffaele Squeri, Alessandro Nicita, Antonio Nirta, Giovanni Genovese, Linda Bartucciotto, Cristina Genovese

**Affiliations:** Department of Biomedical, Dental and Morphological and Functional Imaging Sciences, University of Messina, 98122 Messina, Italy; france.loddo@gmail.com (F.L.); plagana@unime.it (P.L.); caterina.rizzo93@gmail.com (C.E.R.); serenamariacalderone@gmail.com (S.M.C.); b.romeo@hotmail.it (B.R.); roberto.venuto@hotmail.it (R.V.); dmaisano@unime.it (D.M.); f.fedele1965@libero.it (F.F.); squeri@unime.it (R.S.); sandronicita94@gmail.com (A.N.); antonio.n2396@gmail.com (A.N.); lindabartucciotto@gmail.com (L.B.)

**Keywords:** vaccination, gut microbiota, systematic review

## Abstract

**Background**: Vaccination constitutes a low-cost, safe, and efficient public health measure that can help prevent the spread of infectious diseases and benefit the community. The fact that vaccination effectiveness varies among populations, and that the causes of this are still unclear, indicates that several factors are involved and should be thoroughly examined. The “intestinal microbiota” is the most crucial of these elements. Numerous clinical studies demonstrate the intestinal microbiota’s significance in determining the alleged “immunogenicity” and efficacy of vaccines. This systematic review aimed to review all relevant scientific literature and highlight the role of intestinal microbiota in COVID-19, *Salmonella typhi*, *Vibrio cholerae*, and rotavirus vaccinations. **Materials and Methods**: The MESH terms “vaccines” and “microbiota” were used to search the major scientific databases PubMed, SciVerse Scopus, Web of Knowledge, and the Cochrane Central Register of Controlled Clinical Trials. **Results**: Between February 2024 and October 2024, the analysis was conducted using electronic databases, yielding a total of 235 references. Finally, 24 RCTs were chosen after meeting all inclusion criteria: eight studies of COVID-19, two studies of *Salmonella typhi*, three studies of *Vibrio cholerae*, and eleven studies of rotavirus. Only six of these demonstrated good study quality with a Jadad score of three or four. **Conclusions**: According to the review’s results, the intestinal microbiota surely plays a role in vaccinations’ enhanced immunogenicity, especially in younger people. As it is still unclear what mechanisms underlie this effect, more research is needed to better understand the role of the intestinal microbiota.

## 1. Introduction

Due to the wide range of available vaccination platforms, vaccines are currently the most cost–benefit-ratio-efficient weapon in the fight against 33 communicable diseases (CDs). Some forms of these interventions, such as mRNA vaccines, also have applications in the field of oncology in addition to being essential tools in public health [1]. Vaccination, however, causes an immune response that differs widely from person to person, which impacts precision medicine results [2]. Various internal and external factors, including age, genetics, pre-existing immunity, nutritional status, and potential comorbidities, cause different responses to vaccination [3]. Furthermore, it is commonly known that vaccines, especially oral ones, are less immunogenic in developing countries in particular, due to the hygienic and socioeconomic conditions that lead to a higher risk of gastrointestinal infections in developing nations as opposed to Western nations [4,5,6,7]. The gut microbiota comprises several trillion bacterial cells, forming a vast microbial ecosystem essential for developing and controlling immune responses, including reactions to various vaccines [7,8,9].

The intestinal microbiota plays a crucial role in the development and formation of the host’s immune system response [10]. It has several functions, including preventing the growth of pathogens through competitive mechanisms [11], regulating intestinal endocrine processes, and promoting the growth and vascularization of host cells [12,13,14]. Additionally, it controls bone density and neurotransmitter synthesis [15,16], provides 5–10% of the host’s daily energy requirements, and serves as a source of energy biogenesis [17]. Moreover, it facilitates the synthesis of vitamins [18], neurotransmitters [15], and many other substances with unknown targets; it supports the metabolism of bile salts [19] and allows for the elimination of exogenous toxins [20].

Microbes from the *Bacteroides* and *Firmicutes* families contribute to most of the intestinal microbiota of healthy adults, but smaller amounts of *Actinobacteria*, *Proteobacteria*, and *Verrucomicrobia* are also present [21]. *Methanogenic archaea* (primarily *Methanobrevibacter smithii*), *Eucarya* (primarily yeasts), and various phages are also present [22].

Although the intestinal microbiota of adults is more stable than that of newborns at the bacterial phylum level, the relative distributions of subspecies (strains) can be highly unique to each individual. Nonetheless, the functional capacity of the adult gut microbiota is relatively consistent across healthy individuals [11,12,13,14,15,16,17,18,19,20,21,22]. The gut microbiota of elderly individuals typically has a lower variety of bacterial species, which aligns with the decline in immunocompetence associated with aging [23,24,25].

Various endogenous and exogenous factors contributing to childhood obesity [26] and allergies [27] also affect the composition of the intestinal microbiota [28,29]. Other factors include the newborn’s delivery method [30], the host’s genetic traits [31], the host’s immune response [32], dietary habits such as breastfeeding, formula feeding, and the use of food supplements [33], xenobiotics (like antibiotics) and other medications [4,5,6,7,8,9,10,11,12,13,14,15,16,17,18,19,20,21,22,23,24,25,26,27,28,29,30,31,32,33,34], infections [35], circadian rhythm [36], and environmental microbial exposures [37].

The non-gut microbiota, which includes the microorganisms present in other areas of the body such as the skin, respiratory tract, urogenital tract, and breast milk, plays an important role in the immune response to vaccinations.

However, the most important factor regarding the local response to intradermal vaccines like the smallpox vaccine is a robust immune response at the injection site, which is facilitated by antigen-presenting cells. Skin commensal bacteria, such as *Staphylococcus epidermidis*, stimulate the immune system through the recognition of microbial-associated molecular patterns (MAMPs), enhancing the recruitment of immune cells and improving vaccine response. Skin microbiota modulate the activity of Langerhans cells, which are crucial for antigen presentation in intradermal vaccines.

The microbiota of the upper airways, composed of bacteria such as *Streptococcus pneumoniae* and *Haemophilus influenzae*, interacts with the respiratory mucosa, influencing the efficacy of intranasal vaccines (e.g., the influenza nasal spray vaccine). A balanced microbiota promotes the production of secretory immunoglobulin A (IgA), which is essential for mucosal immunity. A healthy respiratory microbiota can enhance the immune response to vaccines targeting respiratory infections (e.g., SARS-CoV-2 or pneumococcal vaccines).

The composition of the oral microbiota affects the response to oral vaccines (e.g., rotavirus or cholera vaccines). Bacteria such as *Streptococcus salivarius* can modulate local immune function and enhance the immunogenicity of ingested vaccines.

While the gut microbiota has been more extensively studied, increasing evidence highlights the influence of site-specific microbiota in modulating vaccine responses.

Based on these premises, a systematic review of the literature was conducted to highlight the role only of the gut microbiota in immune responses to vaccines.

## 2. Materials and Methods

In order to find every clinical study that examined the relationship between intestinal microbiota and vaccinations, we searched the main scientific databases (PubMed, SciVerse Scopus, Web of Knowledge, and Cochrane Central Register of Controlled Clinical Trials) using the following search terms: “microbiota”; “vaccines”; using the “AND” and “OR” functions. We looked through all relevant article bibliographies, including reviews, to find additional references. There were no restrictions on language use. Using a combination of trial searches and collaborative efforts by independent researchers and knowledgeable users, the search terms were developed based on the Medical Thesaurus’s topic heading criteria. We used exclusion criteria to further screen titles, abstracts, or complete articles after removing duplicates. Two authors (F.L. and C.G.) independently carried out the screening. A third author handled any disputes among reviewers regarding eligibility. A data extraction form that was reviewed by the other co-authors was used by the first two authors to extract data from the included papers. The PRISMA standards for reporting systematic reviews are adhered to by these processes [38]. We limited our search to the gut microbiota and its role in the vaccination response.

Search strategy:

(“gut microbiota” OR “intestinal microbiota” OR “gut flora” OR “intestinal flora”)

AND

(“vaccination” OR “vaccine response” OR “immune response” OR “vaccines” OR “vaccine efficacy”)

AND

(“immune system” OR “immunomodulation” OR “immune response” OR “immune function” OR “immune activation”)

AND

(“role” OR “impact” OR “association” OR “influence”)

### 2.1. Data Extraction

After identifying possibly relevant papers, two independent reviewers (F.L. and C.G.) collected the following information: first author’s surname, year of publication, clinical trials, gov identifier (if applicable), study design, the total number of participants, age range, type, disease background, and study arms, with several participants vaccinated in each arm. After this process, we selected only clinical studies to write this article.

### 2.2. Exclusion Criteria

Exclusion criteria ruled out observational studies, systematic reviews, and studies published in a language other than English. Most of the studies were excluded because they did not have a control group. We chose to describe the relationship between the intestinal microbiota and four specific vaccines, because we would have preferred to describe only oral vaccines, and a higher percentage of articles concerned these four vaccines.

### 2.3. Assessment of the Quality of Studies

The Cochrane guidelines for systematic reviews of interventions [38] were applied, and the quality of each study included in the review was independently evaluated by two reviewers (C.G., F.L.). Randomized controlled trials (RCTs) were assessed using the Jadad scale [39]. This assigns an overall methodological quality score ranging from zero to five. Although the studies were not based on this rating, quality scores were used when outlining the findings.

### 2.4. Management of Missing Data

We conducted our analysis based solely on the existing data.

### 2.5. Assessment of Bias Reporting

The assessment of publication bias was not applicable due to the small number of available studies. Publication bias may be present in the review.

## 3. Results

**Figure 1:**
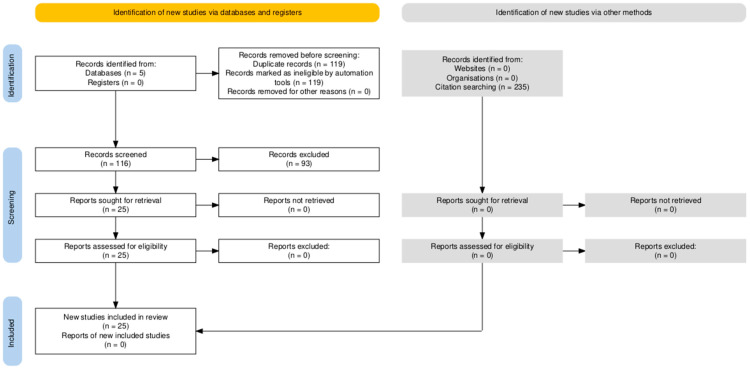
Flowchart of the evaluation and inclusion process [40].

A total of 235 references were found using electronic databases in the February 2024 and December 2024 searches. Once duplicate entries were removed (n = 116), references were reviewed for inclusion based on title and/or abstract. Subsequently, 119 potentially relevant articles were added to the list for full-text assessment. The lack of a control group led to the exclusion of most of these studies. Finally, 24 random clinical trials (RCT) that met our inclusion criteria were selected (see Figure 1).

### 3.1. Quality of the Study

Out of twenty-four clinical trial studies (RCT), we only considered the six (25%) which achieved a Jadad scale score of 3 or 4, which implies a higher level of quality of RCT.

### 3.2. Publication Bias

We also evaluated other studies and grey literature to identify publication bias. We did not perform a funnel plot.

### 3.3. Clinical Trial Studies with COVID-19 Vaccines

A study by L. Daddi et al. [41] investigated the role of the gut microbiota in enhancing the immunogenicity of mRNA vaccines. The research included 16 healthy participants with an average age of 30.4 years. Blood and stool samples were collected, and anti-Spike IgG levels were measured to assess intestinal microbiome composition from baseline (time 0) to one week after the first and second vaccine doses. The effectiveness of the COVID-19 vaccination in patients receiving transplants who are considered non-responders following two doses of the vaccine is the focus of another pertinent study conducted by Julian Singer et al. [42]. Plant-based polysaccharide inulin has been proposed as a means of enhancing vaccination response and managing gut microbiota dysbiosis in this patient population. These results suggest that dietary interventions that modify the gut flora can enhance the effectiveness of vaccination. It has been suggested that inulin, a plant-based polysaccharide, could improve vaccination response and control gut microbiota dysbiosis in this patient group. These findings indicate that dietary interventions, which alter the gut flora, can improve the efficacy of immunization.

Additionally, to explore a potential relationship between the human gut microbiota and metabolic function as well as response to the SARS-CoV-2 vaccine, Bo Tang et al. [43] assessed a group of 200 people. This research suggests that gut bacteria and their activities are linked to a higher response to an inactivated vaccine, the Sinopharm BIBP COVID-19 vaccine (BBIBP-CorV), which could increase the efficacy of COVID-19 vaccinations. Additionally, DoAE raised IgG and IgM antibody levels and the immunomodulatory cytokine IFN-γ. Moreover, compared to the control group, DoAE therapy increased the abundance of anti-inflammatory bacterial species such as *Firmicutes* while drastically reducing the abundance of *Proteobacteria* and pro-inflammatory bacterial species, including *Prevotella*, *Erysipelotrichaceae*, and *Desulfovibrio* [44].

The question of whether older participants who took probiotics before and after the fourth dose of the COVID-19 vaccine would experience longer-lasting vaccine protection has been investigated by Jean-Charles Pasquier [45]. Their immune response may be impacted by the microbial dysbiosis they commonly display. Older adults are known to be more susceptible to COVID-19-related morbidity and mortality for several reasons, including a higher incidence of COVID-19 infection and a more rapid decrease in immune function.

The gut microbiota composition of 138 people who had either received the inactivated CoronaVac vaccine or the BNT162b2 mRNA COVID-19 vaccine was investigated by Siew C. Ng et al. [46]. Blood and stool samples were taken one month following the second dose and at baseline (within three days of the first dose). Serological tests were carried out to evaluate the levels of antibodies in the plasma collected at the beginning of the vaccination cycle and 1 month after the second vaccination dose, and finally, the sequencing of the participants’ microbiota was performed. The study found that BNT162b2 recipients had higher levels of neutralizing antibodies, which correlated with the abundance of flagellated bacteria like *Roseburia faecis*. In contrast, *Bifidobacterium adolescentis* levels were higher in those responding well to CoronaVac, with their microbiome enriched in carbohydrate metabolism pathways. Additionally, *Prevotella copri* and *Megamonas* species were associated with fewer adverse events, suggesting an anti-inflammatory role in the immune response.

Finally, in immunocompromised patients receiving infliximab treatment for intestinal bowel disease, James L. Alexander et al. [47] investigated whether the intestinal microbiota and the metabolome—a collection of all an organism’s metabolites that can participate in its biological processes—can explain the variation in responses to the anti-SARS-CoV-2 vaccination. All study participants received serological testing once every eight weeks. The results showed that a lower variability of the intestinal microbiota’s composition was associated with vaccine responses that were below average. Higher levels of *Streptococcus* were linked to a poorer serological response, while higher gut concentrations of *Bilophila* were linked to a better response. These results point to a connection between gut flora and the different degrees of protection that the COVID-19 vaccine offers to individuals with compromised immune systems. Trimethylamine, one of the metabolites produced by bacteria, might help offset the immunosuppressive effects of anti-TNF treatment.

### 3.4. Clinical Studies on Vibrio cholerae

Yoshikazu Yuki et al. conducted a phase-1 double-blind, randomized, placebo-controlled study [48]. To assess the safety, tolerability, and immunogenicity of the rice-based vaccine MucoRice CTB, which contains the B subunit of cholera toxin (CTB), Japanese men between the ages of 20 and 40 had their serum and feces tested for antibodies against CTB. Following the patients’ division into cohorts, the vaccine was given to them in doses of 1, 3, and 6 g. Results showed that patients receiving higher dosages of Mucorice CTB had higher serum levels of CTB-specific IgG and IgA antibodies without experiencing significant side effects. The increase in neutralizing antibodies against diarrheal toxins was found to be dependent on the intestinal microbiota, with a prevalence of *E. coli* and *Shigella* compared to *Bacteroides*.

Two studies were not included because they are also in progress [49,50].

### 3.5. Clinical Studies on Salmonella

The composition and function of the intestinal microbiota after vaccination and subsequent exposure to wild *Salmonella enterica* serotype *typhi* were analyzed by Yan Zhang et al. [51] to evaluate clinical outcomes. A double-blind placebo study was conducted to assess the impact of vaccination on the makeup and functionality of the gut microbiota using either three doses of the Ty21a vaccine or one dose of the candidate vaccine, M01ZH09. Participants in the study were either healthy male volunteers or non-pregnant females, ages 18 to 60, who had never had a typhoid vaccination or contracted *S. typhi*. Transcripts of *Methanobrevibacter* or another representation of genera in the phylum Firmicutes predominated in the two consistent patterns of gene expression for the human gut microbiota that were found in stool samples collected from research participants. There was no appreciable change in the composition or function of the gut microbiota following immunization against *S. typhi* with either of the two oral live attenuated vaccines. People with the *Methanobrevibacter* transcriptome at time 0 were found to have a lower risk of developing typhoid symptoms after catching wild-type *S. typhi*. According to the findings, the treatment strategy may be impacted by modifications in the gut microbiota’s ion homeostasis and redox potential after exposure to wild-type *S. typhi*. Finally, alterations in the intestinal microbiota composition after exposure to the wild-type *S. typhi* strain were linked to either increased susceptibility to typhoid infection or increased host resistance to it.

A longitudinal study determined whether the licensed oral live attenuated typhoid vaccine, Ty21a, altered the microbiota and whether a particular microbiota composition or subsets thereof are linked to immune responses against *S. typhi* [52]. The composition and temporal evolution of the fecal microbiota in individuals who received the Ty21a typhoid vaccine and those who did not, as controls, were investigated using bacterial 16S rRNA pyrosequencing. The analysis revealed significant intra- and inter-individual heterogeneity, but no obvious alterations to the bacterial complex were observed following the vaccination. Serum anti-LPS IgA and IgG titers were used to identify humoral responses, and multiparametric flow cytometry was used to assess *Salmonella typhi*-specific cell-mediated immune (CMI) responses by measuring intracellular cytokine production. The volunteers were categorized based on the magnitude and kinetics of their answers. People who had multiphasic CMI responses had more diverse and complex microbial communities, but people who were able to produce a positive humoral response had the same microbial composition, diversity, and temporal stability. Most of the more than two hundred operational taxonomic units (OTUs) that were found to distinguish between late and multiphasic CMI responders were assigned to the order *Clostridiales*. The remarkable temporal variability of the gut microbiota and the host’s immunological responses can now be better understood thanks to these data.

### 3.6. Clinical Studies on Rotavirus

Pregnant women in their third trimester were registered in Liverpool, UK (n = 82), Blantyre, Malawi (n = 187), and Vellore, India (n = 395) by Edward PK Parker et al. [53] in this prospective cohort study. The children were routinely vaccinated after giving birth, including two doses of Rotarix^®^ (RIX4414 strain), following the routine national immunization schedule (life weeks 6 and 10 in Malawi and India; life weeks 8 and 12 in the UK). The indicator of vaccine intake was represented by the collection of stool samples starting from the first week of life. In India and Malawi, the presence of maternal rotavirus-specific antibodies in serum and breast milk has been observed to have a negative correlation with ORV response, resulting in the reduction of ORV shedding. Despite maternal antibody levels being comparable to those of other cohorts, ORVs spread in the UK without any inhibition. India and Malawi show a negative correlation between increased microbiota diversity and ORV immunogenicity, suggesting that early microbial exposure could negatively impact vaccine efficacy. It was ultimately demonstrated that the negative correlation between rotavirus-specific maternal antibodies in serum and breast milk, as well as pre-vaccination microbiota diversity, and ORV response in India and Malawi is not present in the UK.

To assess the separate and combined effects of improving WASH (water, sanitation, and hygiene) and improving infant and young child nutrition (IYCF, infant and young child feeding) on effectiveness in preventing stunting and anemia, Ruairi C. Robertson et al. [54] chose a subsample from the “Sanitation Hygiene Infant Nutrition Efficacy (SHINE)” study and conducted a cluster-randomized 2 × 2 study. Using the results of prior studies, infants from the current SHINE cohort were chosen for this investigation if they had at least one available stool sample from the 1- or 3-month visits and one available plasma sample obtained following rotavirus vaccination. IgA typically appears in blood and feces 7–28 days after vaccination or rotavirus infection, suggesting that this is a dynamic period of immunological induction. Consequently, it has been proposed that the immunogenicity of RVV may be influenced by the fecal microbiota immediately before, during, or after vaccination. The investigation of the relationship between entomopathogens and RVV immunogenicity was one of the study’s other goals. An analysis of the data revealed that 34 of the 158 infants with stool samples and anti-rotavirus IgA titers were seroconverts of the RVV.

The newborn microbiome was dominated by *Bifidobacterium longum*. The gut microbiota varied significantly between the initial (≤42 days) and subsequent (>42 days) samples; however, the species composition, alpha and beta diversity, and functional metagenomic features did not differ significantly according to the infection status. The only species linked to an anti-rotavirus IgA titer was *Bacteroides thetaiotaomicron*.

In summary, the study found no correlation between the immunogenicity of RVV in this rural Zimbabwean context and the early life composition or function of the gut microbiota. To investigate the reasons behind oral RVV’s poor efficacy in low-income nations, more research is required.

In a prospective study conducted by Jonathan Fix et al. [55], the author’s main objective was to assess the relationship between the gut microbiome and the response to the oral pentavalent vaccine RotaTeq^®^ (RV5) (Merck, Kenilworth, NJ, USA), which was obtained by reassorting human and bovine rotavirus strains in children from Nicaragua through stool collection and serological evaluation. The intestinal microbiome was then analyzed to determine whether there was a correlation between the relative abundance of different bacterial taxa and seroconversion following vaccination. Before the first dosage of RV5, stool samples were obtained, and 16S rRNA amplicon sequencing was used to determine the composition of the microbiome. In 25 of 45 subjects (55.6%), vaccine seroconversion occurred following the first dose of RV5.

There were no statistically significant variations in microbiota composition between respondents and non-responders. In summary, the study results indicate that the response of infants to oral rotavirus immunization may be slightly influenced by gut microbial taxa.

Three groups of 6-week-old Pakistani infants were compared in terms of the pre-vaccination gut microbiota composition in a case-control study by Vanessa Harris et al. [56]. Using phylogenetic microarray analysis, sufficient, high-quality DNA was extracted from 66 out of 71 fecal samples (93%), allowing for further characterization. Of the 66 newborns, ten (15%) exhibited an IgA > 20 IU/mL and were categorized as Rotarix^®^ (RV1) responders. Ten responder infants’ pre-vaccination microbiota (six weeks) were compared to an ad hoc non-responder group. A positive correlation was found between the RV1 response and an elevated ratio of Gram-negative to Gram-positive bacteria. There was an approximately three-fold increase in the abundance of Proteobacteria linked to *E. coli* and *Serratia*. It is interesting to note that a much larger number of *Proteobacteria*, particularly gamma-*Proteobacteria* linked to *E. coli* and *Serratia*, was again found in the gut microbiome of Dutch infants thought to have high RVV immunogenicity when compared to Pakistani RV1 non-responders. In conclusion, certain innate immune responses can be activated by Gram-negative bacteria, including *Proteobacteria*, by expressing flagella or producing toxic lipophilic acid (LPA). Therefore, *Proteobacteria* or their antigenic cell envelope components may function as natural immunological adjuvants in the investigated community of infants from Pakistan.

To ascertain the role of the infant’s gut microbiota and maternally derived antibodies in establishing the IgA immune reaction to the RV vaccine in high- and low-income countries, Kuladaipalayam Natarajan C. Sindhu et al. [57] conducted an observational study that compared three populations. They enrolled pregnant women in their third trimester who were available for follow-up for at least four months following the baby’s birth. When the third regular immunization dosage was completed, the mother-child pair was monitored until the study’s conclusion. The newborns received two doses of the RV1 vaccine (Rotarix), in addition to other standard vaccines, by the national immunization schedule, except for the birth doses of bacillus Calmette–Guérin (BCG), hepatitis B vaccine, and oral polio vaccine (OPV) (India), which are typically given at birth.

Details of breastfeeding, any previous and current illnesses, and antibiotic use were documented in each sampling.

The primary objectives established by the authors were to determine the relationship between the pre-vaccination levels of maternally produced antibodies specific to RVs (IgG) and the antibody titers (immunogenicity) and seroconversion of the infants four weeks following the administration of two doses of Rotarix^®^ at six, eight, and ten weeks of age. A surrogate for vaccine uptake, RV vaccine shedding would also be evaluated in stool on the seventh day following each vaccination dose.

Identifying whether infants who do not seroconvert following RV immunization have a different microbiota was one of the primary objectives, along with comparing the gut microbial populations of individuals who do and do not seroconvert. Furthermore, the study aimed to assess whether gut microbiota changes are related to infants with biomarkers for intestinal and systemic inflammation and examine the impact of maternal microbiome imprinting on the emergence of child dysbiosis.

Revaccination and fecal microbiome composition were evaluated in a case-control study by Vanessa C. Harris et al. [58] in rural Ghana among 6-week-old infants. The newborns’ microbiomes were then compared to those of 154 Dutch newborns who were deemed healthy and reacted to RVV.

Serum samples were collected before the first vaccination dose, given at 6 weeks of age, and the final vaccination dose, given at 4 weeks of age, to assess anti-RV IgA antibodies and evaluate the differences in microbiota composition between 6-week RVV responders and non-responders.

In parallel, the microbiomes of both responding and non-responding Ghanaian newborns were compared with those of a control cohort of healthy, age-matched Dutch newborns. It was expected that the Dutch infants would respond to RVV even though they did not receive it, as evidenced by extensive clinical trial data showing that Northern European countries have a >90% RVV seroconversion rate.

Increased *Streptococcus bovis* abundance and decreased *Bacteroidetes* phylum presence were shown to be related to response to RVV in Ghanaian RVV responders and non-responders, as well as in Dutch and Ghanaian non-responder infants.

Assessing whether the intestinal microbiota affects the rotavirus vaccine’s immunological effects on healthy adult volunteers is the primary goal of one randomized placebo-controlled trial [59]. The study is still ongoing and further data need to be collected.

The “SUN RCT”, a study by Clare R. Wall [60] that is currently in progress, includes 300 children who have not yet begun to wean. The SUN RCT aims to assess variations in concentrations of infant immune markers and protective antibody responses to oral rotavirus vaccine, as well as whether kumara served as the infant’s first food aid in the development of the infant microbiome. The purpose of the SUN RCT is to determine whether the kumara consumed as a first food contributes beneficially to the development of the intestinal microbiota of newborns who have already received a rotavirus vaccination.

In a case-control study by Edward PK Parker et al. [61] serological analysis revealed no appreciable differences in the diversity or makeup of the 16S bacterial microbiota, and the results indicate that the first dosage of Rotarix© was inhibited by the combined use of OPV, following previous studies.

The authors of a double-blind, randomized controlled trial, Robin P. Lazarus et al. [62], hypothesized that probiotic and zinc supplementation would change gut microbiota and immune responses, thereby increasing the immunogenicity of the rotavirus vaccine. Children that were given probiotics again showed a slight improvement in seroconversion, but not zinc. Sixteen significant adverse events were noted; none of these were connected to the study’s treatments.

A randomized controlled study by Vanessa C. Harris et al. [63] investigated whether microbiota modification could enhance RVV’s immunogenicity. Following randomization, healthy adults received either no antibiotics, narrow-spectrum (vancomycin), or broad-spectrum (oral vancomycin, ciprofloxacin, metronidazole) antibiotics before receiving the RVV vaccination.

Determining the absolute change in serum anti-RV IgA titer levels across randomization groups 28 days post-immunization was the study’s main objective. The purpose of the study was to determine whether altering the microbiome could increase RVV’s immunogenicity. The narrow-spectrum group’s RVV immunogenicity increased after 7 days, even though antibiotics did not affect absolute anti-RV IgA titers. Antibiotics also quickly changed the variety of intestinal beta-bacteria while increasing the amount of RV excreted in feces. The research has demonstrated that the immune system’s reaction to RVV is changed when the microbiota is altered.

## 4. Discussion

### 4.1. COVID-19

The reasons behind the differences in the COVID-19 vaccination’s effectiveness are still unknown. According to recent clinical studies, the gut microbiota may affect a vaccine’s immunogenicity and, consequently, its effectiveness. There may be reciprocal interactions between the gut microbiota and the COVID-19 vaccine, with various microbiota components either increasing or decreasing vaccine effectiveness. Additionally, the intestinal microbiota may be significantly impacted by these vaccines, which could lower the microbiome’s overall population and species diversity. There is various evidence indicating a role of the intestinal microbial population in the immune response, and it has been suggested that a key factor in determining the effectiveness of COVID-19 vaccinations may be the intestinal microbiota [64].

COVID-19 vaccinations may also impact overall health by altering the presence of bacteria linked to diseases and controlling the diversity of the gut microbiota [46,47,65,66,67]. The role of the intestinal microbiota in COVID-19 vaccination has been explained by a variety of theories, such as the natural adjuvant hypothesis, the significance of commensal symbionts, the modification of B cell responses by microbial metabolites, and microbiota-encoded epitopes that might resemble COVID-19 vaccine antigens [67,68]. Activated mucosal-associated invariant T (MAIT) cells are essential for defense, and commensal symbionts against bacterial and viral infections may negatively impact these cells due to their impact on activation, migration, and function [69,70]. This suggests that the lack of commensal symbionts may contribute to the microbiota’s immunomodulation of the MAIT cells. Consequently, MAIT cells would have a reduced capacity to protect against illnesses like SARS-CoV-2 [71]. In response to vaccination, MAIT cells enhance both innate and adaptive immune responses [72]. In conclusion, the lack of commensal symbionts can hurt the activation of the immune system through MAIT cells, reducing the effectiveness of vaccination against COVID-19 both from a quantitative and qualitative point of view, with consequent low levels of protection against infections and diseases.

Another way in which the gut microbiota could influence the effectiveness of COVID-19 vaccination is through so-called “natural adjuvants”. Adjuvants can activate the so-called antigen-presenting cells (APC), favoring the recognition of microbial molecules through the so-called pattern recognition receptors (PRR) such as the Toll-like receptor (TLR) and the NOD-like receptor (NLR) [73]. The interaction through the TLR5 receptor is crucial for the best possible production of antibodies (after vaccination with a non-adjuvanted COVID-19 vaccine), as demonstrated in the case of flagellin [74]. Additionally, by affecting other antigen-presenting cells, the microbiota can strengthen the immune response to vaccinations. For example, the presence of flagellin produced by microbes increases antibody responses to adjuvanted influenza vaccinations [75,76]. Macrophages are required for the vaccine to produce an antibody response, and dendritic cells play no part in this process.

### 4.2. Vibrio cholerae

In Southeast Asia and sub-Saharan Africa, cholera is endemic, with approximately 3 million cases recorded annually [77,78,79,80,81,82,83,84,85,86,87,88]. In this disease, most cases are moderate, but severe cases can be fatal within hours after the onset of symptoms and, finally, 50–70% of untreated patients die [87,88]. Efforts to combat cholera have included the use of oral cholera vaccines (OCVs), which have been applied to both endemic and cholera-naïve populations during epidemics [89,90,91,92,93,94]. Formulations of dead whole-cell *V. cholerae* lacking a recombinant cholera toxin (TC) B component are among the most often used OCVs. These vaccines contain inactivated *V. cholerae* O139 and O1 strains from the Inaba and Ogawa serotypes and require one or more doses to protect adults and children over the age of five [95,96,97]. Even while oral cholera vaccinations have produced some hopeful outcomes, children less than five years old may receive less protection from them [98,99]. The etiopathogenesis of this infection is likely influenced by the intestinal microbiota, which is a significant factor in susceptibility to enteric pathogens [100,101,102,103]. *V. cholerae* colonizes the small intestine during infection and secretes large amounts of TC. It has been demonstrated that components of the gut microbiota mediate susceptibility to *V. cholerae* infection [104,105] and seem to be involved in the recovery of persons afflicted with the virus [35]. Recent research indicates that *V. cholerae* susceptibility is influenced by the microbiota’s composition [105]. Numerous relationships between *V. cholerae* and the gut microbiota have been investigated in recent research. *V. cholerae* inhibits the growth of pathogens by interacting with a range of metabolites originating from the gut microbiota, such as short-chain fatty acids and antimicrobial peptides [106,107]. Furthermore, the amount of secondary and deconjugated bile salts in the intestinal lumen could influence *V. cholerae* colonization. Conjugated bile salts prevent pathogen development and virulence [105,108], whereas secondary bile salts stimulate the production of TC and numerous other virulence factors [109]. The intestinal commensal bacterium *Blautia obeum* produces bile salt hydrolase, which reduces the virulence of *V. cholerae*. Bile salts are deconjugated by this enzyme, which also lowers intestinal concentrations of secondary bile salts [105]. Additionally, during colonization, certain intestinal microbiota components can be inhibited by *V. cholerae*’s type VI secretion system (T6SS), which encourages the growth of intestinal pathogens [110]. *Bacteroides* species are less prevalent in humans infected with *V. cholerae* than in those recuperating from the illness [35]. This suggests that during a *V. cholerae* infection, the gut microbiota’s composition varies. It is still unclear how precisely the intestinal microbiota is changed because of T6SS-induced disease and intestinal metabolic regulation. In summary, little is known about the specific roles of the gut microbiota in infections caused by *V. cholerae*, about how toxins produced by these pathogens during infection modify the microbiota to encourage the spread of the pathogen, and about the involvement of the microbiota more broadly in vaccination-induced immunity. Various hypotheses have been formulated, and from the analysis of the available studies regarding this vaccination, the microbiota may have a role in promoting the effectiveness of the vaccination.

### 4.3. Salmonella

One of the main elements of resistance to enteric pathogen colonization seems to be the gut microbiota [111,112,113]; however, the specific process by which this activity occurs has not been fully clarified [114,115].

An unhealthy diet or the use of antibiotics can cause dysbiosis, or the breakdown of the microbiota, which can lead to infection by a variety of pathogenic organisms, including *Salmonella*. By priming, competing with pathogens for nutrition, modifying the host’s immune system, and specifically targeting other microorganisms with the metabolites it produces, the microbiota prevents colonization [116].

Immunization with Ty21a generates antibodies both in the intestinal mucosa and in serum, and memory B cells against some *S. typhi* antigens (e.g., lipopolysaccharide O, antigen H), as well as a broad range of cell-mediated immunological (CMI) responses [117,118,119,120,121,122].

Studies have demonstrated that CMI responses are characteristic of individuals who reacted to immunization with Ty21a. CMI responses are thought to play an essential part in host defense against these intracellular bacteria through multiple pathways, from cytokine generation to the death of infected cells [119,123,124].

The immunological mechanisms that regulate defense against these pathogens, however, continue to be mostly unclear. It has been demonstrated that the preparation, the dosing schedule—three to four doses are necessary for Ty21a vaccination—and the period of post-vaccination follow-up all significantly affect the vaccine’s overall protective efficacy [123,124].

Different geographical populations of people respond differently to oral vaccinations in terms of effectiveness; this is especially true for more sensitive populations in poorer nations. Indeed, the primary factors thought to contribute to variations in immunization effectiveness include socioeconomic circumstances, host genetics, nutritional state, and exposure to infectious pathogens [125].

One of the least studied factors is the constitution of the intestinal microbiota; studies have shown that specific inhabitants of the microbiota are intimately associated with the development of immunity at the intestinal mucosal level [126].

The current literature appears to support the concept that the intestinal microbiota may help improve vaccination effectiveness. Nevertheless, research on the microbiota and vaccination delivery has not yet been conducted, nor has it been determined whether there is a detectable change in the bacterial flora after vaccination. It will be necessary to understand whether a specific composition of the local microbial community is linked to higher vaccine efficacy.

In conclusion, several clinical studies conducted in underdeveloped nations indicate that the gastrointestinal microbiota may have an impact on a vaccination’s effectiveness [125], and data collected from animal models indicate that altering the gastrointestinal microbiota can improve the effectiveness of a vaccine [127,128,129].

In general, individuals who showed a better immune response to vaccination possessed a microbiota with a higher and more varied composition than individuals who only had a late CMI response to Ty21a.

Ultimately, the immunogenicity of the vaccine, and probably also the efficacy, could potentially be related to the composition of the microbiota of the subject receiving the vaccination.

### 4.4. Rotavirus

*Rotavirus* (RV) is a prominent cause of acute gastroenteritis, but more crucially, it is the most common cause of diarrhea-related death in children globally [130], with Africa and Asia contributing to 95% of all rotavirus deaths.

While *Rotavirus* vaccinations (RVV) can significantly lower RV mortality, their effectiveness is diminished in low-income environments, where they are most required.

Several clinical investigations demonstrate that the RVV vaccination is typically 85–98% efficacious against severe *Rotavirus* gastroenteritis in populations with high socioeconomic status [131,132]. This contrasts with 48% and 39% in South Asia [133] and sub-Saharan Africa [134], respectively.

The underlying causes of this disparity in vaccination efficacy are thought to be complex; however, they are still not completely understood [135]. There are several potential contributing factors, including the placenta’s or breast milk’s inhibitory effect on maternal antibody titers, such as RV-specific IgA derived from breast milk [136,137], the potential interference of concurrent oral vaccinations, like the polio vaccine [138], and the blood group with a specific HLA group [16,139,140,141,142,143,144,145,146,147,148,149,150,151,152,153,154,155,156,157,158].

A recent study showed that changes in the gut microbiota brought on by antibiotics might influence people’s immune response to vaccines [56].

During early childhood, when the immune system and gut microbiota are still developing, oral RV vaccinations are administered.

Children were observed to have the poorest responses in the investigations that were taken into consideration. More specifically, compared to those who responded to treatment, the *Bacteroides* population was higher in Ghanaian children who received the oral rotavirus vaccine [58], but in another study, no noteworthy variations were seen in the gut microbiota of children, this time from southern India, in terms of the amount of *Bacteroides* in the population and how they responded to the oral rotavirus vaccine [61].

Several previously illustrated features highlight the substantial connection that exists between the composition of the gut microbiota and the immunogenicity of oral RV vaccines. As a result, the existing strategy for vaccine production and delivery requires a significant change. Future vaccinations, for instance, might contain in their formulation specific probiotics with an immunomodulatory action towards those bacteria capable of promoting the effectiveness of vaccination in subjects whose composition of intestinal bacteria is lacking in those immunomodulatory bacteria.

Bacterial-derived immunostimulatory molecules capable of improving the immunogenicity of vaccines could be included. Next-generation RV vaccines could be created differently, either by creating non-replicating vaccines or by changing the method of vaccination delivery (e.g., parenterally delivered vaccinations), as oral RV vaccines depend on the replication of attenuated RV contained in the vaccine.

Additionally, it is possible to increase the response to the current oral RV vaccines by administering them immediately after antibiotic therapy, as the altered composition of the intestinal microbiota after antibiotic treatment influences RV replication and produces longer-lasting rotavirus-specific IgA responses.

Several theories have been proposed to explain the discrepancy in oral RV vaccine effectiveness between high- and low-socioeconomic nations, and these theories include the role of the gut microbiota’s constitution.

### 4.5. Limit of Our Study

This systematic review does not consider the contribution of non-gut microbiota (e.g., skin microbiota, respiratory tract microbiota, breast milk microbiota, and urogenital tract microbiota), which could significantly influence the immune response to vaccines, especially for intradermal, intranasal, or oral vaccines.

Some vaccines, such as intranasal ones (e.g., influenza nasal spray) or intradermal ones (e.g., smallpox vaccine), are strongly influenced by the local mucosal or skin microbiota, and ignoring these factors can thus lead to incomplete conclusions.

Individual differences in non-gut microbiota (e.g., respiratory microbiota in contexts of high pollutant exposure) can significantly affect vaccine efficacy and are not considered. Conditions of dysbiosis in the respiratory, vaginal, or skin microbiota can compromise the immune response to vaccines either independently or synergistically with the gut microbiota.

A holistic approach that integrates the role of all microbiota is essential to obtain a more complete picture and translate findings into meaningful clinical interventions.

## 5. Conclusions

The various studies discussed above demonstrate a clear correlation between the immunogenicity of the above-mentioned vaccines and the prevalence of specific subsets of gut bacteria. These findings indicate that the microbiota plays a significant role in shaping immune responses to vaccination, with certain bacterial populations being linked to stronger antibody production and better overall immune function. However, most of the research conducted so far has primarily focused on general alterations in the gut microbiota following vaccination, looking at broader shifts in microbial composition and their impact on the immune system. While this provides valuable insights, there is still much to be understood about the precise mechanisms through which the microbiome influences vaccine efficacy. To further improve the effectiveness of currently available vaccines—or potentially guide the development of new ones—future microbiota studies could benefit from a more targeted approach, identifying specific bacterial strains or bacterial-derived molecules that directly modulate the immune response as potential “immune enhancers” by either promoting T-cell activation or antibody production or mitigating inflammatory responses. By isolating and characterizing these beneficial bacteria or bacterial metabolites, researchers could pave the way for targeted microbiota-based interventions—such as probiotic supplements, prebiotics, or dietary strategies—that might be used in conjunction with vaccination to enhance its effectiveness. Current studies have established a connection between the gut microbiome and vaccine responsiveness; future research should aim to identify the exact bacterial strains or microbial products that exert an immune-modulating effect. Such insights could ultimately lead to novel, microbiome-based strategies to boost vaccine efficacy, particularly for immunocompromised populations or those less responsive to traditional vaccine formulations.

## Data Availability

Data are contained within the article and Supplementary Materials.

## Supplementary Materials

The following supporting information can be downloaded at: https://www.mdpi.com/article/10.3390/vaccines13030306/s1, PRISMA 2020 Main Checklist [159].
